# Comparative performance of ChatGPT, Gemini, and Deepseek on endodontic exam questions in Turkish and English

**DOI:** 10.1186/s12903-026-07753-5

**Published:** 2026-02-04

**Authors:** Eda Gürsu Şahin

**Affiliations:** Faculty of Dentistry, Department of Endodontics, Cankiri Karatekin University, Cankiri, Türkiye

**Keywords:** Artificial intelligence, ChatGPT, Dental education, Deep learning, Endodontics, Large language models

## Abstract

**Background:**

Large language model-based artificial intelligence (LLM-based AI) applications have become a focal point in the healthcare field. This study aimed to compare the performance of ChatGPT-4, Gemini 2.0 and DeepSeek-R1 in answering endodontics questions from the dentistry specialty examination in both Turkish and English.

**Methods:**

A total of 130 multiple-choice Endodontics questions from the dentistry specialty examination question pool were presented to LLMs developed by OpenAI (ChatGPT-4), Google (Gemini 2.0) and DeepSeek (DeepSeek-R1). The questions were entered into each model under standardized conditions in both English and Turkish. The responses and their explanations were classified based on predefined criteria as “correct answer and explanation”, “correct answer with incorrect explanation” and “incorrect”.

The R programming language was used within the RStudio environment for statistical analysis. McNemar’s Chi-squared test with continuity correction was applied to analyze the models’ performance in providing correct answers and explanations across different languages, as well as to compare performance between models. Fisher’s Exact Test was used to analyze the models’ responses to different question types. The threshold for statistical significance was set at *p* < 0.05.

**Results:**

When analyzed individually, DeepSeek-R1, Gemini 2.0 and ChatGPT-4 provided correct answers at a higher rate in English compared to Turkish. In Turkish, the performance of DeepSeek-R1 and Gemini 2.0 in providing correct answers and accurate explanations was significantly higher than that of ChatGPT-4. All models demonstrated significantly better performance on Simple-style questions compared to Combination-style questions in both languages.

**Conclusions:**

These findings indicate that LLMs show promise in standardized tests within dentistry. However, despite their ability to recognize patterns and organize data, they have limitations in fully understanding the underlying concepts of information. The results also highlight the need for continuous improvements to enhance their effectiveness across different subjects and languages, as well as the potential occurrence of hallucinations in their responses.

**Supplementary Information:**

The online version contains supplementary material available at 10.1186/s12903-026-07753-5.

## Background

Artificial Intelligence (AI), defined as the simulation of human intelligence by machines, is rapidly transforming fields like healthcare through its capacity to manage and analyze large datasets [1]. Its applications range from disease diagnosis to data interpretation and it shows promise in enhancing dental education by solving complex problems through systematic reasoning. In endodontics, AI applications have significantly expanded, particularly in enhancing diagnostic precision through automated root canal morphology identification and radiographic lesion detection, which are critical for successful clinical outcomes [2, 3].

Natural Language Processing (NLP), enabling computers to understand human language, improves human-computer interaction. Large Language Models (LLMs), which leverage NLP for tasks like machine translation, have become central in NLP research [4]. Models such as ChatGPT and Gemini process vast datasets to generate text but perform less effectively in low-resource languages due to limited training data [5]. Challenges include high costs of data creation and the scarcity of standardized resources as seen in regional Indonesian languages [5].

ChatGPT’s use in healthcare raises concerns about patient privacy, legal risks, and misinformation. Patients may over trust AI, unaware of its limitations, which can affect the physician-patient relationship and treatment adherence. Therefore, assessing the accuracy and reliability of its responses is essential [6].

To address data scarcity, multilingual NLP often employs cross-lingual transfer, using other languages via methods like pivoting, transfer learning or joint training which enhance performance [5]. “Pivoting” uses an intermediate language to enable translation between low-resource languages, while “transfer learning” applies knowledge from high-resource tasks to low-resource ones. Both have shown effectiveness in multilingual NLP tasks [7, 8].

LLMs support scientific writing, literature review and personalized learning, potentially complementing traditional education [9]. They offer real-time feedback and exam simulation, aiding in critical exam preparation [10, 11].

Released in 2023–2025, ChatGPT-4, Google Gemini 2.0, and DeepSeek-R1 have been recognized as transformative tools in dental education. These models, with features such as reinforcement learning (DeepSeek), multimodal learning (Gemini), and Chain-of-Thought reasoning (ChatGPT), offer flexibility and accessibility, especially in resource-limited healthcare settings [12–14]. The key architectural features, release timelines, and multilingual capabilities of the evaluated large language models are summarized in Table 1.

**Table 1:** Comparative overview of the large language models

Model	Developer	Release Date		Architecture	Multilingual Support
ChatGPT-4	OpenAI	March 2023	Transformer-based (Closed)		Extensive
Gemini 2.0	Google DeepMind	December 2024	Native Multimodal Transformer		Extensive (Native)
DeepSeek-R1	DeepSeek	January 2025	Mixture-of-Experts (MoE)		Broad

Use of public LLMs for health-related queries is increasing, as they aid clinical reasoning and decision-making [15]. Their competence in global medical exams, such as the United States Medical Licensing Examination (USMLE) and national exams in Japan and China, demonstrates their ability to process complex information [16]. ChatGPT-4 has passed some professional exams but also raised concerns about accuracy and performance in languages other than English [17, 18]. The model has gained popularity in dental education, assisting in personalized learning, article writing and test creation [3, 19, 20]. In endodontics, previous studies have explored the use of AI for root canal morphology detection, radiographic interpretation, and clinical decision support, demonstrating its potential to enhance diagnostic accuracy and educational outcomes [2, 21]. However, AI’s impact on academic integrity and education remains uncertain. Despite their usefulness in information retrieval, LLMs may hallucinate (producing incorrect outputs with high confidence) highlighting the need for caution [22].

Limited research exists on LLM performance in national exams, especially in Turkey, and no studies currently address DeepSeek-R1 in dental education, underlining a research gap. The Turkish Dental Specialty Examination (DUS) consists of 120 multiple-choice questions based on the courses completed during undergraduate dental education, including 40 questions from the Basic Sciences test and 80 questions from the Clinical Sciences test. Of the 80 Clinical Sciences questions, approximately 12.5% are Endodontics questions.

This study evaluated ChatGPT-4, Gemini 2.0, and DeepSeek-R1 by testing their responses to questions from Turkey’s dental specialization exam in Endodontics. The Dental Specialty Examination is taken by dentists seeking postgraduate specialization and by candidates who have completed undergraduate dental education and are eligible for professional practice [23].

The central aim of this study was to evaluate the performance, reliability, and potential hallucinations of LLMs in the context of dental education. The null hypothesis posited no significant differences in performance among models or between languages. Specifically, we investigated whether answer justifications could reveal hallucinations and assess model reliability. By analyzing both Turkish and English responses, this study addresses a critical gap in the literature regarding the applicability and effectiveness of AI models in multilingual dental education, thereby providing evidence on their potential role as educational tools and assessment aids.

## Methods

### Data selection of endodontics questions

This research was designed as an in-silico study to evaluate the performance of LLMs (ChatGPT-4; Gemini 2.0; DeepSeek-R1) on Endodontics questions. Therefore, the data used in the study included Endodontics questions from the national dentistry specialization exams held in Turkey between April 2012 and October 2021. Since 10% of the questions were officially shared between 2022 and 2024, only the Endodontics questions and answer keys of these years that were publicly available were included. The questions and answers are publicly accessible on the official website.

A total of 140 questions were initially included; questions that had been officially annulled by the examination authority due to inaccuracies or incompleteness were excluded from the evaluation. For validity purposes, image-based questions were also excluded. A total of 130 questions were used for further analysis.

### Question categorization

**Fig. 1:**
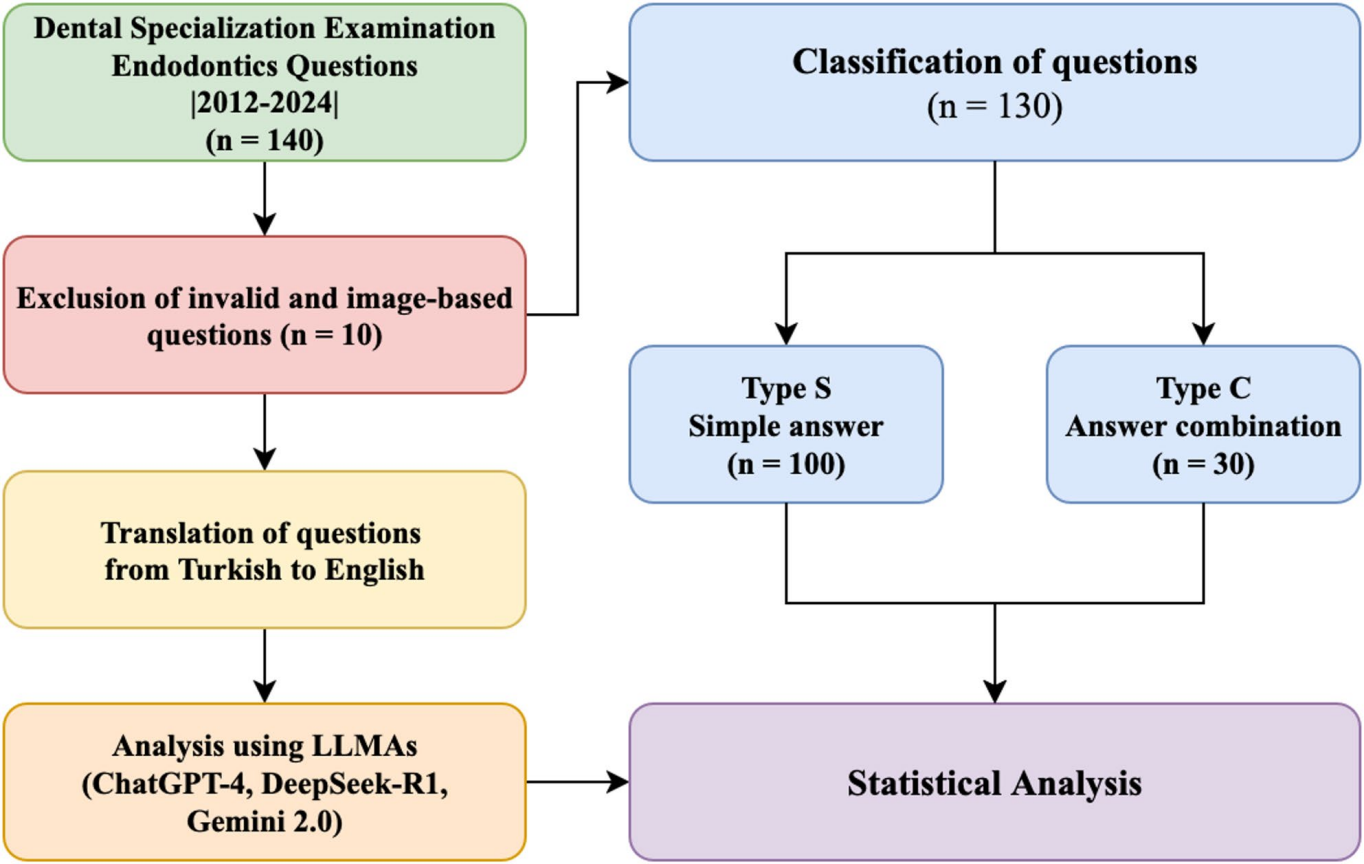
Flow Chart showing the overview of the study design. n, number of questions; LLM; Type S, question in simple answer style; Type C, question in answer combination style

The questions were categorized into two subcategories. Type C questions (answer combination, *n* = 30) required selecting the correct answer option containing the right combination of statements related to the question. Type S questions (single answer, *n* = 100) required selecting a single correct answer statement (for a flowchart, see Fig. 1). Due to structural differences and their potential impact on LLM performance, an additional analysis distinguishing between question types was conducted. Since the analysis was performed in two languages, the questions were translated into English by the first author (DDS, fluent in English) and terminology was adapted using Systematized Nomenclature of Dentistry (SNODENT) [24]. The translations were then reviewed by a professional with expertise in medical translation and academic editing to ensure accuracy and consistency. Any discrepancies during the translation process were resolved through discussion and consensus between the first author and the medical translation expert.

### Prompt design

The prompting for the LLMs was conducted between January 31 and February 2, 2025, with each model–language condition completed in a single continuous session. The prompt period was kept to a minimum. All questions were entered one by one by a researcher, first in Turkish and then in English, using the official web interfaces of the LLMs (https://chatgpt.com/, https://Chat.DeepSeek-R1.com, https://gemini.google.com/app).

**Fig. 2:**
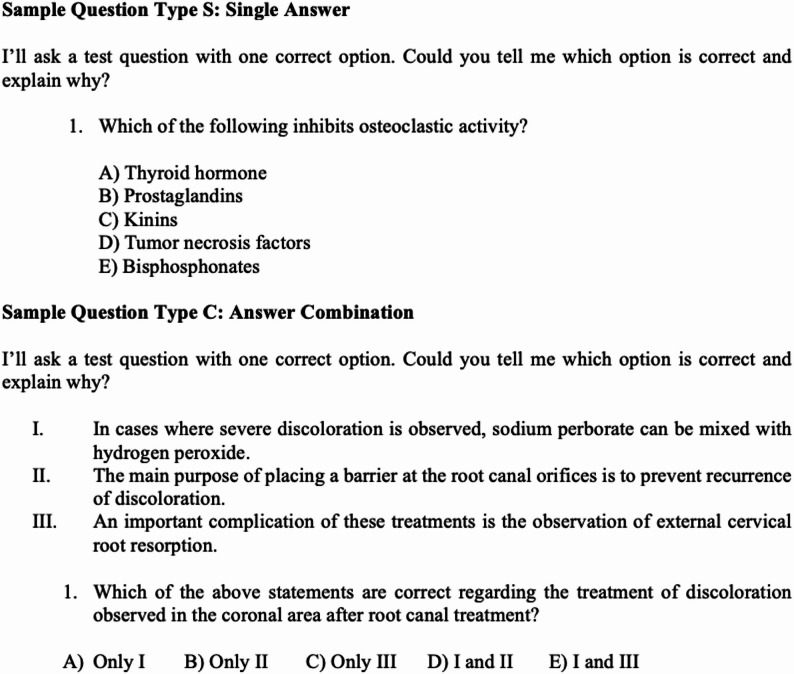
Example question prompt types used for evaluation. Quantitative questions were entered into the LLM using the same prompt but with different answer choices. For Type C questions, the LLM was required to select an answer choice that corresponded to the given statements. Type S questions directly prompted the LLM to choose the correct answer for the given question

To ensure methodological transparency and reproducibility, each question was submitted only once with a standardized prompt, and the same questions were presented to all models under identical conditions. Model responses were generated using the default inference settings of each platform; parameters such as temperature or stochastic decoding could not be manually controlled and were therefore kept consistent by relying on standardized prompts and single-response generation. Prompt engineering can affect the output results, so a single prompt was used for each question to ensure the quality and standardization of the results: “I’ll ask a test question with one correct option. Could you tell me which option is correct and explain why?” Examples of the prompt and questions entered in English are shown below (see Fig. 2). The same prompt was used for the Turkish entries.

Before entering a new question, the previous conversation was cleared and a new session was started. This approach minimized the chances of context retention or in-context learning influencing the results. Each question was entered only once during the analysis.

### Evaluation method

All responses and explanations from the LLMs were categorized as: ‘correct answer and explanation’, ‘correct answer but incorrect explanation’ and ‘incorrect’. The answers were recorded in a Microsoft Excel (version 16.88) spreadsheet. Based on the answers provided by the models, performance results were analyzed by question type and language.

As the official answer key provided only the correct option without explanatory justification, the accuracy of explanations was determined based on clinical and scientific correctness through joint evaluation by two experienced academic endodontists, with reference to standard textbooks when needed. Explanations containing clinically misleading, scientifically inaccurate, or incomplete information were classified as incorrect.

### Statistical analysis

The answers and explanations provided by each model in both Turkish and English were recorded in a Microsoft Excel file. The data was then analyzed using RStudio (version 2024.12.0.467) [25] with the R programming language [26]. Additionally, the *ggplot2* [27] package was used for data visualization.

For within-model comparisons of correct answers and correct explanations across languages, McNemar’s Chi-squared test with continuity correction was used. For comparing model performances based on different question types, Fisher’s Exact Test was applied. The statistical significance level was set at *p* < 0.05.

## Results

**Fig. 3:**
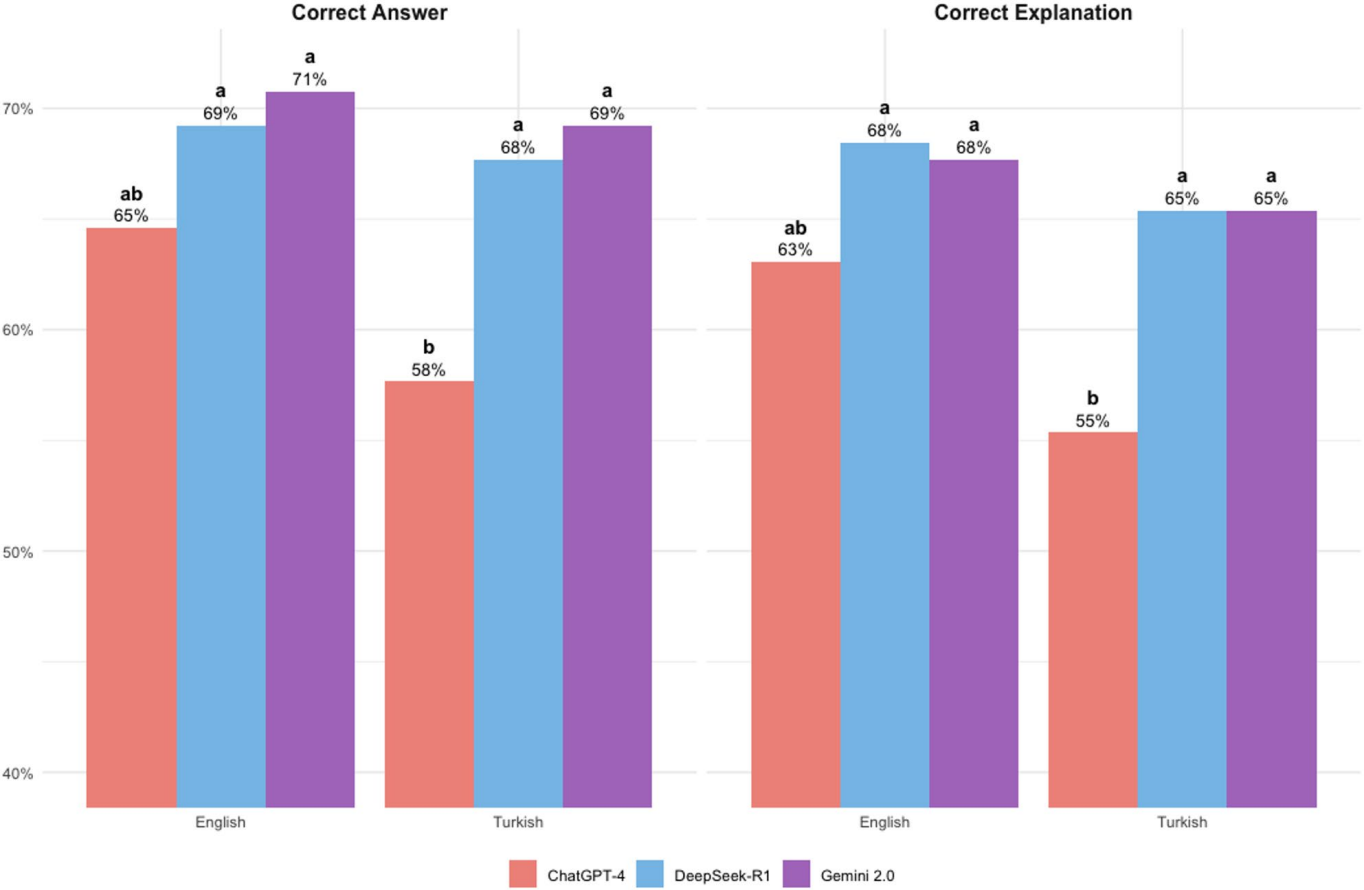
Comparison of answer and explanation accuracy in English and Turkish across different LLMs. Group stacked bar chart representing the accuracy rates of answers and explanations for each LLM in Turkish and English languages. Significant differences between the LLMs are indicated with different letters

Figure 3 presents the performance of LLMs in providing correct answers and correct explanations in Turkish and English.

In Turkish: DeepSeek-R1 performed statistically better than ChatGPT-4 in terms of both correct answers (*p* = 0.0425) and correct explanations (*p* = 0.0425). Gemini 2.0 also outperformed ChatGPT-4 in correct answers (*p* = 0.0093) and correct explanations (*p* = 0.0311).

In English: Although the descriptive ranking of models for correct answers and explanations was Gemini, followed by DeepSeek and ChatGPT, no statistically significant differences were found between the models (*p* > 0.05).

When comparing Gemini and DeepSeek, their performance in both languages was very close and no statistically significant difference was found (*p* > 0.05).

Within-model comparison: While numerical scores were higher in English than in Turkish for all models, these differences did not reach statistical significance (*p* > 0.05). 

**Fig. 4:**
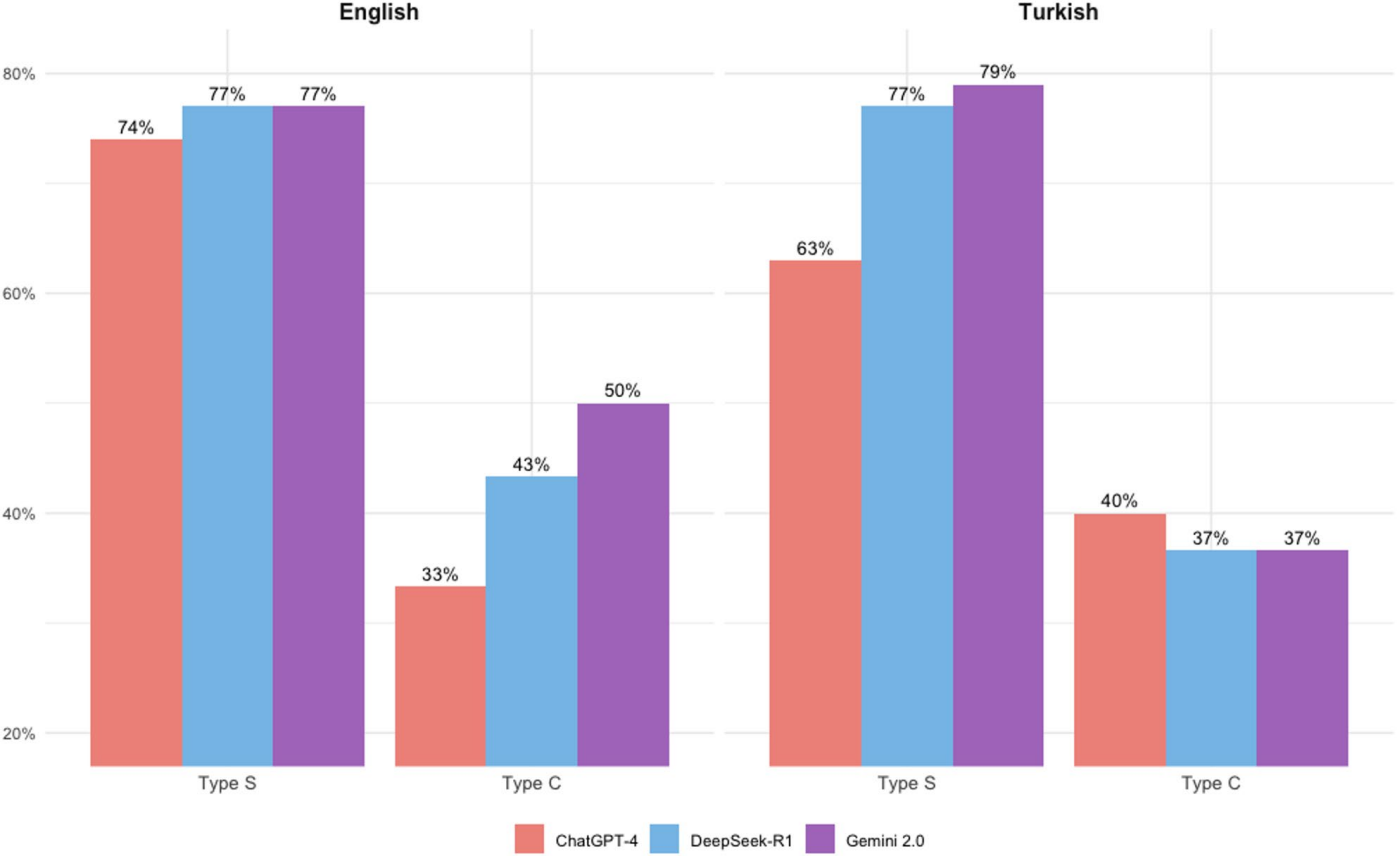
Comparison of answer accuracy for type S and C questions in English and Turkish across different LLMs. Group stacked bar chart representing the accuracy rates of answers of Type S and Type C questions for each LLM in Turkish and English languages

Figure 4 presents the performance analysis of LLMs based on Type C and Type S question styles in Turkish and English. All models performed better on Type S questions than on Type C questions in both languages (*p* < 0.01). Only in the ChatGPT-4, the difference in providing correct explanations between Type S and Type C questions in Turkish was found to be marginally significant (*p* = 0.0618). The *p*-values for this analysis are presented in Table 2.

**Table 2:** Comparison of answer and explanation accuracy rates for different question types and *p*-value results of fisher’s exact tests

Model	Language	Question Type	Correct Answer (% [*n*/*N*])	*p*-value	Correct Explanation (% [*n*/*N*])	*p*-value
ChatGPT	English	Type S	74% [74/100]	< 0.001	72% [72/100]	< 0.001
		Type C	33% [10/30]		33% [10/30]	
	Turkish	Type S	63% [63/100]	0.0347	60% [60/100]	0.0618*
		Type C	40% [12/30]		40% [12/30]	
DeepSeek	English	Type S	77% [77/100]	0.0012	76% [76/100]	0.0073
		Type C	43% [13/30]		43% [13/30]	
	Turkish	Type S	77% [77/100]	< 0.001	76% [76/100]	< 0.001
		Type C	37% [11/30]		30% [9/30]	
Gemini	English	Type S	77% [77/100]	0.0062	74% [74/100]	0.0074
		Type C	50% [15/30]		47% [14/30]	
	Turkish	Type S	79% [79/100]	< 0.001	74% [74/100]	< 0.001
		Type C	37% [11/30]		37% [11/30]	

* Close to the significance threshold (marginally significant).

## Discussion

To the best of our knowledge, this is the first study to evaluate the performance of DeepSeek-R1 in answering multiple-choice questions in endodontics and to compare LLM outputs in both Turkish and English, as no prior research was found on this topic in PubMed, MEDLINE, Scopus, or Google Scholar up to the date of this study. Beyond identifying correct answers, the study also examined whether explanations included misleading information, addressing the risk of hallucinations. In endodontics, AI applications have significantly expanded, particularly in enhancing diagnostic precision through automated root canal morphology identification and radiographic lesion detection, which are critical for successful clinical outcomes [2, 3].

The evaluated questions, sourced from the endodontics section of Turkey’s national dental specialization exam, reflect real exam content rather than artificially created items, enhancing the study’s relevance. However, since the questions are country-specific, results should be interpreted with consideration of differing national educational frameworks. Observed differences in LLM performance should also be interpreted in light of variations in question types (single-choice vs. combination-type), language translation (Turkish vs. English), model versions, and testing conditions, which limit direct comparisons with prior studies.

Although LLMs are considered proficient in translation, both prompts and questions were presented in English and Turkish to increase generalizability and reduce translation bias. Analyses were conducted separately for each language, addressing the research gap on Turkish-English performance differences [5]. However, broader studies across diverse clinical and educational contexts are still needed.

The absence of statistically significant intra-language differences and the higher overall performance in English support the notion that these models are well-trained in translation [5]. While performance among models was similar in English, Gemini 2.0 and DeepSeek-R1 outperformed ChatGPT-4 in Turkish, indicating that language remains a relevant factor influencing model performance. DeepSeek-R1’s high Turkish accuracy rate (68%) supports its reported strength in multilingual tasks [28, 29].

In this study, ChatGPT-4 achieved a 65% accuracy rate in English, surpassing the 57% obtained by ChatGPT-3.5 in a previous endodontics study [29]. Furthermore, ChatGPT-4 reached 74% accuracy on single-choice questions in English, an improvement over the 63% reported for ChatGPT-3.5 in earlier research. For combination-type questions, ChatGPT-4 scored 33%, aligning with prior findings [30]. Overall, all three LLMs exceeded 70% accuracy on simple-type questions in English, whereas the highest score on combination-type questions was 50% (Gemini 2.0). In Turkish, simple-type question accuracy surpassed 60% across models, while the best performance on combination-type questions was 40% (GPT) (see Fig. 4). These results suggest that LLMs face significant challenges in processing complex combination-type formats [31].

Although LLMs can detect patterns and structure data, they still face challenges in grasping contextual and semantic depth [32]. This study analyzed a relatively limited number of questions (*n* = 130); however, this sample size is comparable to prior research focusing on a single dental specialization. Unlike broader question banks spanning multiple disciplines, this study focused exclusively on endodontics, which has been less frequently examined in prior LLM performance studies [33].

LLMs present limitations such as potential “hallucinations” and the inability to learn from past interactions [12]. A strength of this study lies in its evaluation of the accuracy of explanations. Despite using a restrictive prompt to minimize fabricated content, the analysis revealed that correct answers were often accompanied by inaccurate explanations, regardless of language (see Fig. 3). While new sessions were initiated to reduce interference, the potential influence of continuous learning on subsequent performance remains an area for further investigation [33].

Future research should explore longitudinal changes in LLM performance on endodontic examination questions. Comparative studies conducted in Japan and other medical fields have reported accuracy rates similar to those observed in the present study, supporting the external consistency of our findings [34–36].

As LLMs continue to develop, higher performance results are expected, as evidenced by studies comparing successive model versions [34, 37, 38].

In a study comparing ChatGPT-O1 and DeepSeek-R1 on critical pediatric clinical cases, DeepSeek-R1 was found to have a lower accuracy rate; nonetheless, it was highlighted as an accessible solution due to its open-source structure. This feature makes DeepSeek-R1 potentially useful in resource-limited healthcare environments or academic projects requiring flexible tools [13].

The variation in LLM performance across different medical and dental subspecialties remains a subject of active discussion. Literature suggests that this discrepancy may stem from the insufficient comprehensiveness of dental education data in training sets and limited public access to specialized dental question banks. This potential “accessibility bias” is often attributed to the smaller student population and narrower scope of institutionalization in dentistry compared to medicine. Enhancing the accessibility of dental data within training datasets is proposed as a key factor in improving LLM performance in this field [39].

While LLMs show potential, significant improvements in reliability are necessary before integration into clinical practice. A critical concern is “automation bias,” where users may over-rely on LLM-generated outputs despite their inherent risks. ChatGPT, Gemini, and DeepSeek can generate seemingly logical but incorrect answers, often referred to as hallucinations, because they are limited by training data that may not include the latest evidence. Moreover, these models may perpetuate biases present in their training data, which underscores the need for continuous data updates and bias-mitigation strategies to ensure accuracy in academic and research contexts [40].

The phenomenon of LLMs providing confident but incorrect answers, or even fabricated references, poses a significant risk in dentistry [41, 42]. This is particularly concerning when information about specific populations or rare conditions is lacking. Therefore, while LLMs serve as valuable assistants in dental education and data mining, they cannot currently replace traditional educational models. Finally, economic barriers to accessing advanced LLMs must be considered. In the current study, ChatGPT-4, DeepSeek-R1, and Gemini 2.0 were evaluated using their free versions to reflect real-world accessibility. Future development should focus on making these advanced tools more accessible to a broader user base, especially in regions with limited financial resources.

### Limitations and future directions

This study has several limitations. First, image-based questions were excluded, and the sample size was below the ideal threshold for robust comparisons. Second, biases in LLM training data may affect their performance on specialized subject areas. The study’s Turkey-specific focus limits generalizability. Third, clinical decision-making in endodontics involves more than multiple-choice questions, including medical history, physical exams, and diagnostic tools. The high accuracy observed in simple multiple-choice questions should be interpreted with caution, as performance significantly declined in combination-type questions. This suggests that the model’s clinical reasoning and educational potential may be more limited than its overall scores imply.

Although the translations were independently reviewed by a medical translation expert, formal intercoder reliability metrics were not calculated for the bilingual translation process, which should be considered a limitation.

The models’ performance is based on data from February 2025 and may change with future updates. LLM effectiveness varies across different dental topics, requiring a careful application. Future research should develop methods to evaluate LLMs in multilingual environments and improve their capabilities in specialized dental fields. Although LLMs show promise, they must be carefully managed, particularly in non-English contexts. Addressing identified biases is crucial for improving their global applicability. Since each question was asked only once, potential variability in LLM responses could not be assessed.

Future studies should also explore LLM performance on visual-based questions. Modified visual inputs could help evaluate their suitability for dental exams.

## Conclusion

This study evaluated the performance of ChatGPT-4, Gemini 2.0, and DeepSeek-R1 on endodontic specialization exam questions in Turkish and English. Gemini 2.0 and DeepSeek-R1 demonstrated comparable high performance, both outperforming ChatGPT-4. While all models excelled in simple-type questions, their success significantly declined in combination-type questions, highlighting limitations in complex clinical reasoning. Despite a strong correlation between correct answers and explanations, the persistent presence of hallucinations suggests that these tools are not yet fully reliable. Consequently, while LLMs offer significant potential as supportive tools, they must be used with caution and critical evaluation in dental education.

## Supplementary Information

Supplementary Material 1.

## Data Availability

The dataset used and analyzed during the current study is available from the corresponding author on reasonable request.

## Abbreviations

AI: Artificial Intelligence
LLM: Large Language Model
NLP: Natural Language Processing
SNODENT: Systematized Nomenclature of Dentistry
USMLE: United States Medical Licensing Examination
