# Supplementary material for: Comparative performance of ChatGPT, Gemini, and Deepseek on endodontic exam questions in Turkish and English
Source: BMC Oral Health. 2026 Feb 4;26:399. doi: 10.1186/s12903-026-07753-5 (PMC12949502; doi:10.1186/s12903-026-07753-5)
Supplement: Supplementary file 1 — Supplementary Material 1. [file 12903_2026_7753_MOESM1_ESM.docx]

1. In cases where severe discoloration is observed, sodium perborate can be mixed with hydrogen peroxide.
2. The main purpose of placing a barrier at the root canal orifices is to prevent recurrence of discoloration.
3. An important complication of these treatments is the observation of external cervical root resorption.
4. Which of the above statements are correct regarding the treatment of discoloration observed in the coronal area after root canal treatment?
5. Only I B) Only II C) Only III D) I and II E) I and III
6. Which of the following is not used to remove old root canal fillings?

A) Rotary shaping systems

B) Manual root canal files

C) Heated instruments

D) Chemical solvents

E) Methylene blue

1. Standard preparation method
2. Step-back method
3. Step-down method
4. Crown-down method
5. Which of the above mentioned root canal preparation methods are applied from coronal to apical?

A)I ve II

B) III ve IV

C) II ve IV

D) I ve III

E) I, II ve IV

I. Provides better working length control.

II. Provides better filling of canal irregularities and accessory canals.

III. Less chance of apical overflow of obturation material.

1. Which of the above are the advantages of the hot vertical compaction technique compared to the lateral condensation method?
2. I ve II
3. I ve III
4. Only I
5. Only II
6. I, II ve II
7. Which of the following is not a factor in fracture of a tooth that has undergone root canal treatment?

A) Excessive expansion during root canal treatment

B) Decrease in the water content of the dentin after root canal treatment

C) Excessive expansion in the coronal part of the canal

D) Excessive compaction pressure during root canal filling

E) Excessive pressure during post placement in the canal

1. Which of the following microorganisms is the most commonly isolated from root canals in unsuccessful endodontic treatments?

A) Enterococcus faecalis

B) Candida albicans

C) Actinomyces israelii

D) Prevotella intermedia

E) Bacteroides melaninogenicus

7. Which of the following statements regarding bacterial biofilms is incorrect?

A) Planktonic bacteria that adhere to a surface play a role in the formation of biofilms.

B) The biofilm structure is resistant to external factors.

C) Bacteria in a biofilm can transfer their genetic characteristics to each other.

D) Planktonic bacteria are more virulent than bacteria in a biofilm.

E) Bacteria in a biofilm can easily survive even in the absence of nutrients

8. Which of the following inhibits osteoclastic activity?

A) Thyroid hormone

B) Prostaglandins

C) Kinins

D) Tumor necrosis factors

E) Bisphosphonates

9. Which of the following is the angle formed between the cutting edge and the long axis of the canal file?

A) Cutting angle

B) Helix angle

C) Rake angle

D) Orientation angle

E) Groove angle

10. Which of the following is the most effective combination for revascularization and disinfection of the environment in regenerative endodontic applications?

A) Streptomycin / Tetracycline, (1:1)

B) Penicillin / Clarithromycin, (1:1)

C) Ciprofloxacin / Metronidazole / Minocycline, (1:1:1)

D) Cephalosporin / Tetracycline / Sulfonamide, (1:1:1)

E) Penicillin / Erythromycin, (1:1)

11. Which of the following is not a resin-based root canal filling material?

A) RoekoSeal

B) Epiphany

C) AH26

D) Diaket

E) EndoREZ

12. Which of the following does not play a role in the success of vital pulp treatment options in the treatment of complicated crown fractures?

A) Treatment within the first 24 hours following trauma

B) Ensuring a bacterial-tight seal

C) Using the fractured crown fragment in treatment

D) Using a pulp coating agent with high antibacterial properties

E) Choosing calcium hydroxide or mineral trioxide aggregate as a pulp coating agent

13. After root end surgery, without angiogenesis, adequate blood supply to the area cannot be provided and wound healing cannot be completed.

Which of the following is not one of the angiogenesis stimulatory factors?

A) Vascular endothelial growth factor

B) Transforming growth factor-α

C) Interleukin-1

D) Tumor necrosis factor-α

E) C-terminal “cross-linking” telopeptide

14. Which of the following is not a benefit of the irrigation solutions used in root canal treatment?

A) Dissolving organic debris

B) Blocking dentinal tubules

C) Eliminating microorganisms

D) Wetting the canal walls

E) Providing a disinfection effect in areas where root canal instruments cannot reach

15. Which of the following nerve fibers is responsible for pain transmission in the pulp?

A) A-alpha

B) A-beta

C) A-delta

D) A-gamma

E) B

1. Carbamide peroxide

II. Hydrogen peroxide

III. Sodium chlorite

IV. Sodium perborate

16. Which of the above substances are not used in intracoronal bleaching?

1. Only III
2. Only IV
3. I ve II
4. II ve IV
5. III ve IV

17. Which of the following irrigation solutions has organic tissue solvent properties?

A) Chlorhexidine

B) Formaldehyde

C) Sodium hypochlorite

D) Ethylene diamine tetra-acetic acid

E) Iodine potassium iodide

18. Which of the following statements is incorrect for the clinical and radiographic examination of internal root resorption in permanent teeth?

A) The location of the radiolucent area does not change when radiographs are taken from different angles.

B) In radiographic examination, there is no deterioration in root canal continuity.

C) In radiographic examination, there is no destruction on the outer surface of the root.

D) If radiographically the resorption has not reached the external surface of the root, there is no resorption in the adjacent bone.

E) The vitality test usually gives a positive response.

19. Which of the following is the current treatment regarding the type and duration of splinting in horizontal root fractures?

Type - Duration

A) Rigid splinting 2-4 days

B) Rigid splinting 2-4 weeks

C) Rigid splinting 2-4 months

D) Semi-rigid splinting 2-4 weeks

E) Semi-rigid splinting 2-4 months

20. Which of the following is a “solid core” carrier system used in canal filling?

A) System B

B) Inject – R Fill

C) Ultrafil

D) ThermaFil

E) Obtura

21. Which of the following is not a rotary instrument system used in root canal shaping?

A) EndoSequence

B) Self-Adjusting File System

C) K3

D) RaCe

E) System B

22. A patient who presented within 24 hours after the trauma was found to have a complicated crown fracture in the upper central tooth with incomplete root development. The radiograph showed that there was no root fracture. Which of the following is the most appropriate treatment for this patient?

A) Root canal treatment

B) Partial pulpotomy

C) Total pulpotomy

D) Apexification

E) Apical resection

23. When does cementogenesis begin at the root tip after a successful apical resection?

A) 5-6 months

B) 3-4 months

C) 2-3 months

D) 10-12 days

E) 2-4 days

24. Which one of the following is not one of the tests used to determine pulp vitality?

A) Thermal test

B) Electric pulp test

C) Anesthetic test

D) Laser Doppler Flowmeter test

E) Cavity test

25. Which of the following destroys all microorganisms except spores when disinfecting endodontic instruments?

A) Quaternary ammonium compounds

B) Phenolic compounds

C) Iodoform

D) Benzalkonium chloride

E) ​​Glutaraldehyde

26. Which of the following is not an advantage of rubber dam application during endodontic treatment?

A) Protecting the patient

B) Protecting the clinician

C) Facilitating the determination of the working length

D) Preventing contamination of the root canal

E) Increasing the visibility of the working area

27. Which of the following collagen types are found more in the pulp tissue?

A) Type I-Type III

B) Type II - Type IV

C) Type II - Type V

D) Type II - Type VI

E) Type III -Type VII

28. In which of the following endodontic infection conditions is antibiotic use unnecessary?

A) Systemic infections with fever

B) Cellulitis

C) Osteomyelitis

D) Chronic infections with fistulization

E) Persistent infections

29. Which of the following is not secreted by odontoblasts?

A) Type I collagen

B) Cysteine

C) Acid phosphatase

D) Dentin sialoprotein

E) Alkaline phosphatase

30. Which of the following does not play a role in the initiation of inflammatory reactions in the pulp?

A) Histamine release

B) Bradykinin release

C) Activation of plasma proteins

D) Parathyroid hormone release

E) Degranulation of mast cells

31. Which of the following approaches should be applied in vertical root fractures?

A) Root canal treatment

B) Extraction

C) Splinting

D) Trepanation

E) Root amputation

32. Which of the following is not a root canal shaping technique?

A) Balanced force technique

B) Standardized technique

C) Active step-up technique

D) Step-back technique

E) Crown-down technique

33. Laser is not used for which of the following in endodontics?

A) Cleaning and disinfection of canals

B) Obturation of root canals

C) Endodontic retreatment

D) Apical surgery

E) Detection of canals that cannot be localized

34. Which of the following is not one of the bacterial transmission routes that lead to "endodontic periodontal combined" lesions?

A) Pulp Stone

B) Dentinal Tubules

C) Accessory Canals

D) Apical Foramen

E) Palatogingival Grooves

35. Which of the following is not a cause of fracture of rotary root canal instruments?

A) The tip of the instrument gets stuck in the canal and the instrument continues to rotate, creating a torsional type fracture

B) Increasing axial pressure

C) Intense pus flow from the canal

D) Forcing excessive bending

E) Metal wear due to corrosion and rust

36. The electric pulp test used to measure the vitality of teeth is used to stimulate which of the following nerve fibers?

A) A-alpha

B) A-beta

C) A-delta

D) B

E) C

37. Which of the following statements is incorrect regarding preparation with nickel-titanium rotary instruments?

A) Before preparing with rotary instruments, it is necessary to work up to the number 20 handpiece.

B) They can break after long-term use in narrow and curved canals.

C) By using rotary instruments with passive tips, the formation of steps in the canal is tried to be minimized.

D) Low pressure should be applied when using rotary instruments.

E) The step created with stainless steel instruments in the canal can be easily overcome with rotary instruments

38. Which of the following is not a change in an aging pulp?

A) Innervation

B) Immune Response

C) Vascularity

D) Cell Number

E) Apical Foramen diameter

39. Which of the following increases the effectiveness of irrigation solutions used in root canal treatment?

A) Reducing the amount of solution

B) Reducing the concentration of the solution

C) Cooling the solution

D) Activating the solution with ultrasonic devices

E) Long time elapsed since preparation

40. Which of the following is not one of the aids used in removing gutta percha from the canal?

A) Masserann set

B) Ultrasonic instruments

C) Rotary instruments

D) Chemical solvents

E) Application of heat

41. Which of the following does not help in the diagnosis of vertical fractures?

A) Complaint of pain during chewing

B) Radiography

C) Electric pulp test

D) Staining methods

E) Probing

42. Which of the following does not cause mobility in the tooth?

A) Acute pulpitis

B) Chronic trauma

C) Periodontal diseases

D) Root fractures

E) Acute trauma

43. At which of the following should the root canal preparation be terminated anatomically?

A) Minor apical foramen

B) Radiological apex

C) Within Black space

D) Major apical foramen

E) 1 mm beyond radiological apex

44. Which of the following describes the anticurvature technique used in root canal shaping?

A) Standard preparation

B) Removal of less dentin from thin dentin walled areas of the root in curved canals

C) Removal of equal dentin from each wall of the root canal

D) Shaping the root canal from apical to coronal

E) Shaping without beveling the canal file

45. Which of the following is not one of the methods that facilitates the detection of the canal entrance during root canal treatment?

A) Using an operating microscope or magnifying glasses

B) Using dyes such as methylene blue in the pulp chamber

C) Exploring the pulp chamber floor with instruments such as probes

D) Monitoring the color changes seen in the pulp chamber

E) Narrow opening of the access cavity

46. ​​Which of the following is not a finding of acute apical abscess?

A) Swelling

B) Spontaneous pain

C) Inability to observe the apical change radiographically

D) Fistulization and occasionally pus drainage

E) Sensitivity to pressure on the tooth

47. Which of the following irrigation solutions, when used together, results in the formation of a toxic precipitate called parachloroaniline?

A) Chlorhexidine gluconate and ethylenediamine tetraacetic acid

B) Ethylenediamine tetraacetic acid and hydrogen peroxide

C) Sodium hypochlorite and chlorhexidine gluconate

D) Citric acid and hydrogen peroxide

E) ​​Sodium hypochlorite and ethylenediaminetetraacetic acid

48. Which of the following is not one of the layers of the pulp?

A) Odontoblast layer

B) Pulp proper layer

C) Cell-rich layer

D) Reticular layer

E) Cell-poor layer

49. The tip diameter (D0) and working end diameter (D16) of a 20-numbered 0.02 taper file manufactured according to ISO standards are given in which of the following?

A) D0: 0.20 mm, D16: 0.36 mm

B) D0: 0.20 mm, D16: 0.52 mm

C) D0: 0.02 mm, D16: 0.18 mm

D) D0: 0.02 mm, D16: 0.34 mm

E) D0: 0.20 mm, D16: 0.32 mm

50. Which of the following collagen types is most abundant in the organic matrix of dentin?

A) Type I

B) Type II

C) Type III

D) Type IV

E) Type V

51. Which of the following statements is incorrect regarding the properties of chlorhexidine used as a root canal irrigation agent?

A) It is effective against gram (+) and gram (-) bacteria

B) It has a bacteriostatic effect at low concentration and a bactericidal effect at high concentration

C) It is absorbed by dental tissues and mucosa, thus increasing its effect with long-term release.

D) It is an agent that effectively removes the smear layer.

E) It affects the cell wall of the microorganism and increases the permeability of the membrane, thus providing an antibacterial effect.

52. Which of the following is not used in thermal tests, which is a method used to determine pulpal vitality?

A) Ethyl chloride

B) Carbon dioxide snow

C) Tetrafluoroethane

D) Dichlorodifluoromethane

E) Urethane dimethacrylate

53. Intracoronal tooth whitening procedures cause which of the following root resorption?

A) Internal

B) Apical

C) Lateral

D) Cervical external

E) Replacement

54. After avulsion, which of the following is the order of most preferred to least preferred waiting solutions that can be used until the tooth is reimplanted;

I. saliva,

II. physiological serum,

III. water,

IV. milk

Which of the following is the order of the soaking solutions from most preferred to least preferred?

1. II – IV – I – III
2. II – IV – III – I
3. IV – I – II – III
4. IV – I – III – II
5. IV – II – III – I

55. Which of the following is not one of the causes of intrinsic (internal) discoloration in teeth?

A) Erythroblastosis fetalis

B) Calcific metamorphosis

C) Intrapulpal hemorrhage

D) Tatracycline use

E) Chromogenic microorganisms

56. Which of the following is not a neuropeptide found in pulp nerve cells?

A) Calcitonin gene-related peptide

B) Substance P

C) Neuropeptide Y

D) Neurokinin A

E) Nerve growth factor

57. Which of the following is one of the microorganisms that survives extraradicularly in apical tissues and is associated with failure after root canal treatment?

A) Propionibacterium propionicum

B) Enterococcus faecalis

C) Porphyromonas endodontalis

D) Fusobacterium nucleatum

E) Prevotella nigrescens

58. Which of the following is not a cause of endodontic flare-up?

A) Hyperocclusion

B) Excessive weakening of canal walls during post space preparation

C) Pushing of dentinal and pulpal debris into the periapical region

D) Incomplete removal of pulp tissue

E) Canal preparation beyond the apical end point

59. Which of the following tests is used to determine the dental arch from which the pain originates?

A) Biting test

B) Cavity testing

C) Selective anesthesia testing

D) Vitality testing

E) Dyeing test

A 26-year-old pregnant woman with a history of root canal treatment comes to the clinic in the afternoon. Radiography is not taken from the patient during the diagnosis and treatment stages. It is learned that the patient is in the 6th month of her pregnancy and articaine-containing local anesthetic is used during her treatment. The working size is determined with an apex locator device and the treatment is performed while the patient is in a supine position. The root canals are filled after preparation.

60. Which of the following is one of the procedures performed incorrectly during treatment?

A) Not using radiography

B) Position of the patient

C) Performing this treatment in the 6th month of pregnancy

D) Preferred local anesthetic

E) Using an apex locator

61. Which of the following irrigation solutions has the longest lasting antibacterial effect on dentin?

A) Sodium hypochlorite

B) EDTA

C) Chlorhexidine

D) Citric acid

E) Hydrogen peroxide

62. Which of the following is not used as a root-end filling material?

A) IRM

B) Glass ionomer cement

C) Diaket

D) MTA

E) MTAD

63. Which of the following post types provides bonding to the root canal walls with adhesive bonds, distributes the incoming forces throughout the tooth structure and minimizes the risk of fracture?

A) Zirconia posts

B) Titanium posts

C) Fiber posts

D) Stainless steel prefabricated posts

E) Cast posts

64. Which of the following clinical tests is not used to evaluate the presence of a crack in a tooth?

A) Bite test

B) Percussion test

C) Staining

D) Laser doppler flowmeter test

E) Transillumination

65. Which of the following is not one of the natural scaffolds?

A) Bioceramics

B) Glycosaminoglycan

C) Demineralized dentin matrix

D) Collagen

E) Fibrin

66. How many types of root canal configurations can be encountered in permanent teeth according to the Vertucci classification?

1. 5
2. 6
3. 7
4. 8
5. 9

67. Which of the following agents are included in the triple antibiotic paste mixture used in root canal medication?

A) Metronidazole+Bacitracin+Ampicillin

B) Metronidazole+Minocycline+Ciprofloxacin

C) Metronidazole+Doxycycline+Ampicillin

D) Metronidazole+Chloramphenicol+Penicillin

E) Metronidazole+Penicillin+Trimethoprim-sulfamethoxazole

68. A radiolucent area related to the lower left first premolar tooth is detected in serial periapical films taken from a 40-year-old male patient who applied for routine control. The tooth, which is not accompanied by any restoration or carious lesion, is determined to be vital as a result of the electric pulp test and the pocket depth measurement is within normal limits. No sensitivity is detected in the percussion and palpation tests and it is determined that there is no mobility and marginal bone loss in the tooth. Accordingly, which of the following is the most probable clinical picture present in the relevant tooth?

A) Chronic apical periodontitis

B) Acute apical abscess

C) Hyperemia

D) Periapical cemental dysplasia

E) Acute pulpitis

69. In an adult patient, an uncomfortable sensation, sensitivity or pain in the tooth as a result of the “bite test” cannot be caused by which of the following?

A) Periradicular periodontitis

B) Crack in the tooth

C) Cusp fracture

D) Condensing osteitis

E) Fractured, wide, multifaceted coronal filling

70. What is the D16 diameter of the 0.02 mm diameter increase number 25 K-file from standardized root canal instruments used manually in endodontics?

A) 0.25

B) 0.41

C) 0.57

D) 1.05

E) 1.25

71. Which of the following statements about sodium hypochlorite, one of the irrigation solutions used in endodontics, is incorrect?

A) It has very good antibacterial properties.

B) It is a good necrotic and vital tissue solvent.

C) It completely removes the smear layer.

D) The volume used and the duration of contact with the tissue are important in the success of root canal treatment.

E) Tissue solvent effectiveness increases with increasing temperature.

72. Which of the following retrograde filling materials has the potential to stimulate periapical healing?

A) Amalgam

B) Glass ionomer cement

C) Composite resin

D) Mineral trioxide aggregate

E) Zinc oxide eugenol cement

73. When using posts in root canals, what should be the minimum length of canal filling material left apically to prevent apical leakage?

A) 4-5

B) 6-7

C) 8-10

D) 11-13

E) 14-16

74. Which of the following is not a local hemostatic agent that can be used during endodontic surgery?

A) Ferric sulfate

B) Calcium sulfate

C) Thrombin

D) Atropine

E) Bone wax

75. I. Decrease in dentin permeability

II. Formation of repair dentin

III. Inflammation of the pulp connective tissue on the surface closest to the dentin

IV. Movement of fluid in the dentin tubules

Which of the above are the reactions of the pulp-dentin complex to protect the pulp against caries?

A) Only I

B) I and II

C) II and III

D) I, II and III

E) II, III and IV

76. Which of the following is a developmental anomaly characterized by the development of symptomatic periapical lesions, usually seen in premolars, after the tooth has fully erupted into the mouth and is in full contact with its antagonist?

A) Taurodontism

B) Dens Evaginatus

C) C-shaped canal

D) Radix entomolaris

E) Gemination

77. Infection from a mandibular tooth does not spread to which of the following regions?

A) Buccal vestibule region

B) Submandibular region

C) Submental region

D) Sublingual region

E) Infraorbital region

78. A 35-year-old male patient who complained of severe pain in his upper right posterior tooth was found to have a carious lesion involving the pulp in his upper right first molar tooth on periapical radiography. No pathological findings were found at the root apex and the lamina dura was observed to be healthy. It was learned from his history that the pain started spontaneously and that the patient was constantly taking painkillers. Severe and prolonged pain was detected as a result of cold application to the relevant tooth. No sensitivity was detected in percussion and palpation tests. Which of the following is the most probable clinical picture and treatment method for this patient?

A) Hyperemia - Capping treatment

B) Acute pulpitis - Root canal treatment

C) Chronic apical periodontitis - Root canal treatment

D) Acute apical abscess - Capping treatment

E) Periapical cemental dysplasia - Root canal treatment

79. Which of the following statements about electric pulp testing (EPT) is incorrect?

A) The response of the pulp to EPT does not provide information about histological health or disease status.

B) EPT should be applied to the incisal third of anterior teeth and the middle third of posterior teeth.

C) In young patients with teeth that have not completed root development, cold tests are more reliable than EPT.

D) In ​​healthy teeth with EPT measurement, the threshold value response is higher in anterior teeth than in posterior teeth.

E) The most appropriate location for EPT measurement is the tooth surface adjacent to the pulp horn.

80. Which of the following is not a factor affecting the duration and intensity of dental anesthesia?

A) Slow or rapid injection of the anesthetic solution

B) Individual patient reaction to the anesthetic material

C) Condition of the tissue to be anesthetized

D) Technique used to provide anesthesia

E) Anatomical variations of the area to be anesthetized

81. A 30-year-old female patient who complained of sensitivity in her upper left posterior tooth was diagnosed with a limited carious lesion in the dentin of her upper left first molar tooth on a periapical radiograph. No pathological findings were found at the root apex and there was no loss of laminadura. Her history shows that she had not taken any painkillers for the sensitivity in her tooth, that the sensitivity was provoked by a cold drink and did not last long. It is learned that there was no spontaneous pain. Vitalometric examination shows that the tooth is vital and that there is no sensitivity in percussion and palpation tests.

Which of the following is the most likely diagnosis for this patient?

A) Acute apical periodontitis

B) Acute pulpitis

C) Chronic apical periodontitis

D) Hyperemia

E) Acute apical abscess

82. Which of the following is not used to remove the smear layer?

A) Tetracycline isomer, acid and detergent mixture

B) Maleic acid

C) Chlorhexidine digluconate

D) Ethylenediaminetetraacetic acid

E) Citric acid

83. In which of the following is the use of the plastic core carrier root canal filling technique contraindicated?

A) Tooth with excessive curvature of the canal

B) Tooth with long canal

C) Tooth with thin roots

D) Tooth with open apex

E) Tooth to be post-restored

84. Which of the following is the most common cause of increased tooth fragility after endodontic treatment?

A) Decrease in the water content of the tooth due to loss of vitality after endodontic treatment

B) Long-term intracanal drug application with calcium hydroxide

C) Canal irrigation using high concentration (6%) sodium hypochlorite

D) Occlusal access cavity preparation itself

E) Inclusion of both margins in the access cavity preparation in addition to the access cavity preparation

I. There are more nerve fibers in the tubules of root dentin than in the coronal dentin tubules.

II. The number of A-δ fibers in the pulp is greater than the number of A-β fibers.

III. Substance P is secreted in neurogenic inflammation.

85. Which of the following statements are true about the nerve tissue of the pulp?

A) Only I

B) Only III

C) I and II

D) II and III

E) I, II and III

A systemically healthy 9-year-old child is brought in one hour after the trauma that occurred after a blow to his right upper central incisor. Clinical and radiological examinations reveal that the pulp horn is exposed to a diameter of approximately 1 mm due to a crown fracture in the relevant tooth. It is seen that the tooth, which has not yet completed its root development, maintains its vitality, and it is determined that there is no displacement in the tooth.

86. Which of the following is the most appropriate treatment for this patient?

A) Apexification treatment

B) Revascularization treatment

C) Root canal treatment

D) Direct capping treatment

E) Tooth extraction

I. Late response to vitality test

II. Radiolucent image at the apex of the tooth

III. Provoked pain

IV. Spontaneous pain

87. Which of the above are clinical findings of reversible pulpitis?

A) Only I

B) Only III

C) I and IV

D) II and III

E) II and IV

88. The occlusal surface of the permanent maxillary first molar tooth resembles which of the following shapes?

a) Square

b) Rhomboid

c) Rectangle

d) Triangle

e) Oval

89. Which of the following materials is not used in vital pulp treatments?

a) Calcium hydroxide

b) Resin modified glass ionomer cement

c) Calcium silicate based cements

d) Epoxy resin based cements

e) Mineral trioxide aggregate

90. Which of the following types of stem cells does not contribute to the regeneration of the pulp-dentin complex in revascularization therapy?

a) Apical papilla stem cells

b) Pulp stem cells

c) Periodontal ligament stem cells

d) Bone marrow mesenchymal stem cells

e) Adipogenic stem cells

91. Which of the following is not a method used to localize the entrance of a root canal whose entrance cannot be detected?

a) Checking the pulp chamber floor with a sharp-ended flame-tipped bur

b) Locating the canal orifice with a sharp-tipped probe

c) Staining the pulp chamber with 1% methylene blue

d) Taking radiographs from different angles and cone-beam computed tomography

e) Creating a bubble by holding NaOCl in the pulp chamber

I. Fistula mouth

II. Ecchymosis

III. Lymphadenopathy

92. Which of the above can be seen both inside and outside the mouth as a result of examination methods?

a) Only I

b) Only II

c) Only III

d) I and II

e) I, II and III

I. Calcium hydroxide

II. Ethylenediamine tetraacetic acid

III. Chlorhexidine digluconate

93. Which of the above agents can be used to dissolve necrotic and vital pulp tissue during endodontic treatment in a patient known to be allergic to sodium hypochlorite?

a) Only I

b) Only II

c) I and III

d) II and III

e) I, II and III

94. Which of the following materials is not included in the American Association of Endodontists' current regenerative endodontic treatment protocol?

a) Calcium hydroxide

b) Antibiotic paste

c) 1.5% NaOCl

d) 17% EDTA

e) 2% chlorhexidine digluconate

95. A 45-year-old patient presents with a complaint of sharp and severe pain during chewing in the right mandibular region. A crack is suspected in the mandibular right permanent first molar tooth with a large amalgam restoration. Which of the following approaches is less decisive than the others in diagnosing a crack in this patient?

a) Pain monitoring after orthodontic band application

b) Taking Periapical radiograph

c) Bite test

d) Application of dye solution after removal of filling material

e) Examination using transillumination and magnification tools

I. Reduces the risk of root fracture.

II. Allows the permanent restoration of the tooth to be completed earlier.

III. Can provide the formation of a biological hard tissue at the root tip.

96. In the treatment of teeth with open apex and necrotic pulp; When the apical barrier technique performed with MTA and calcium hydroxide apexification are compared, which of the above are the advantages of using MTA?

a) Only I

b) Only III

c) I and II

d) II and III

e) I, II and III

I. Facultative bacteria are dense.

II. Oxygen pressure is low.

III. Carbohydrates are the main food source.

IV. The amount of bacteria is higher than coronal.

97. Which of the above statements are correct regarding the apical root canal ecosystem in advanced root canal infections?

a) I & III

b) I & IV

c) II & III

d) Only II

e) Only IV

I. May cause dark gray discoloration in vital teeth.

II. May make root canal localization difficult.

III. Only seen after the fifth decade.

98. Which of the above statements are correct regarding pulp calcifications?

a) Only I

b) Only II

c) Only III

d) I and II

e) II and III

99. Which of the following causes a false positive response in the electric pulp test?

a) Inadequate isolation of the tooth

b) Calcification of the root canal

c) The root of the tooth has not completed its development.

d) Use of drugs that increase the pain threshold

e) Having recently experienced dental trauma

100. In a tooth with an open apex diagnosed with reversible pulpitis, it is determined that the pulp is exposed during cavity opening. Which of the following is the most appropriate treatment approach for this tooth?

a) Conventional endodontic treatment

b) Endodontic treatment after creating an apical barrier with MTA

c) Endodontic treatment after apexification treatment with Ca(OH)2

d) Pulp capping or partial pulpotomy

e) Revascularization treatment

101. Which of the following internal bleaching applications is safer than the others for preventing external cervical root resorption?

a) Sodium perborate and 30% hydrogen peroxide mixture without heat activation

b) Sodium perborate and 35% hydrogen peroxide mixture without heat activation

c) Sodium perborate and water mixture without heat activation

d) 35% carbamide peroxide with heat activation

e) 30% hydrogen peroxide with heat activation

I. Mineral trioxide aggregate

II. Calcium hydroxide

III. Dual antibiotic paste (ciprofloxacin, metronidazole)

102. Which of the above materials are used in regenerative endodontic treatment and may cause discoloration in the relevant tooth?

a) III only

b) I only

c) I and II

d) II and III

e) I, II and III

103. Which of the following is the second step of the balanced force technique, which is one of the root canal expansion techniques?

a) The file, which is placed in the canal without applying pressure, is rotated 90° clockwise with light apical pressure.

b) The file is rotated 360° clockwise without applying pressure.

c) With sufficient apical pressure, the file is rotated 180-270° counterclockwise.

d) The file is rotated 180° clockwise with a little more pressure towards the apex.

e) The file is moved 90° counterclockwise and then rotated 180° clockwise.

I. It has no antibacterial effect.

II. It is used to reveal growth factors trapped in root dentin in regenerative endodontic treatments.

III. Its pH value is between 2-3 and effectively removes organic materials from the root canal.

104. Which of the above statements about ethylenediamine tetraacetic acid (EDTA) are correct?

a) Only I

b) Only II

c) Only III

d) I and II

e) II and III

I. Reimplanting an avulsed tooth as soon as possible has a positive effect on the prognosis of the tooth.

II. The most appropriate time to start root canal treatment in an avulsed tooth with complete root tip development is one month after the tooth is reimplanted.

III. Rigid splints are preferred to ensure stability after an avulsed tooth is reimplanted.

105. Which of the above statements are correct regarding traumatic injuries resulting in avulsion?

a) Only I

b) Only II

c) Only III

d) I and II

e) I and III

I. Vital pulp treatments

II. Treatment of open apex teeth

III. Retrograde filling

106. Which of the above are common areas of use of calcium hydroxide and calcium silicate based materials in endodontics?

a) Only I

b) Only III

c) I and II

d) II and III

e) I, II and III

I. Salivary components

II. Necrotic pulp tissue

III. Dentin collagen

107. Which of the above can be used as a food source by microorganisms settled in the root canal system?

a) Only II

b) I and II

c) I and III

d) II and III

e) I, II and III

108. Which of the following statements is incorrect regarding the histopathological findings of a tooth with internal root resorption?

a) Multinucleated giant cells are observed within the resorption lacunae.

b) The protective odontoblast layer and predentin on the root canal wall are damaged, and the underlying mineralized dentin tissue is exposed.

c) An increase in the number of cells and the amount of collagen in the pulp connective tissue can be observed.

d) Inflammatory cells are observed in the pulp connective tissue.

e) In a tooth with active internal root resorption, the pulp tissue apical to the resorption area is necrotic.

109. Which of the following statements about odontoblasts is false?

a) Tight junctions prevent the passage of some substances between odontoblasts.

b) Synthesizes the phosphophorin protein that participates in extracellular mineralization.

c) Synthesizes type I and type V collagen.

d) It is the most numerous cell type in the pulp.

e) Its number per unit area is higher in the coronal pulp than in the root pulp.

I. Hydrogen peroxide

II. Sodium hypochlorite

III. Chlorhexidine digluconate

110. Which of the above irrigation solutions can adversely affect resin bonding to root canal dentin?

a) I only

b) II only

c) III only

d) I and II

e) II and III

I. Maxillary 2nd premolars are more likely to have a single root and two canals than a single root and single canal.

II. Mandibular 2nd premolars are mostly single root and single canal.

III. Mandibular laterals are more likely to have a single root and two canals than a single root and single canal.

111. Which of the above statements about root canal anatomy are correct?

a) Only I

b) Only III

c) I and II

d) II and III

e) Only II

112. Which of the following statements about dental pulp stem cells is incorrect?

a) They play a role in reparative dentinogenesis.

b) They are postnatal mesenchymal stem cells.

c) They can differentiate into neurons under appropriate conditions.

d) They are found in the cell-rich layer of the pulp.

e) They are unipotent cells.

113. Which of the following microorganisms is least likely to be seen in primary intraradicular infections?

a) Porphyromonas gingivalis

b) Candida albicans

c) Fusobacterium nucleatum

d) Porphyromonas endodontalis

e) Treponema denticola

I. Tooth with symptomatic irreversible pulpitis

II. Tooth with asymptomatic apical periodontitis

III. Tooth with condensing osteitis

114. Which of the above are suitable for completing root canal treatment in a single session?

a) Only I

b) Only II

c) I and III

d) II and III

e) I, II and III

115. Which of the following statements are true regarding the intraligamentary injection technique that can be used to provide pulpal anesthesia?

I. Pulpal anesthetic effect begins 10-15 minutes after injection.

II. Pulpal anesthetic effect lasts at least 45 minutes.

III. It is a type of intraosseous anesthesia technique.

a) Only I

b) Only II

c) Only III

d) I and II

e) I and III

116.Which of the following dose/time recommendations is not appropriate for antibiotic prophylaxis in an adult patient with a history of infective endocarditis who is scheduled for root canal treatment?

a) 2000 mg Amoxicillin orally, 1 hour before the procedure

b) 2000 mg Ampicillin IM or IV, 30 minutes before the procedure

c) 600 mg Clindamycin orally, 1 hour before the procedure

d) 2000 mg Azithromycin orally, 1 hour before the procedure

e) 2000 mg Cephalexin orally, 1 hour before the procedure

117. Which of the following is not one of the factors that cause sodium hypochlorite solution to overflow into periapical tissues during endodontic treatment?

a) Excessive pressure applied during irrigation

b) Iatrogenic widening of the apical foramen

c) Irrigation needle stuck in the canal

d) Presence of perforation

e) Presence of apical delta

118. The outer diameter of the smallest Gates Glidden bur is the same as the apical end of which ISO standard root canal instrument?

a) 30

b) 40

c) 50

d) 60

e) 70

I. Use with EDTA

II. Use with etidronic acid

III. Use by heating to 60 °C

119. In which of the above cases does the organic tissue solubility of sodium hypochlorite increase?

a) Only I

b) Only III

c) I and II

d) I and III

e) II and III

I. Gutta-percha

II. Radiopaque agents

III. Zinc oxide

IV. Plasticizing agents

120. Which of the following is the order of the materials found in gutta-percha from most to least?

a) III - I - II - IV

b) I - III - II - IV

c) I - II - III - IV

d) III - IV - I - II

e) II - IV - III – I

I. Irrigate the root canal with saline solution

II. Apply local anesthesia

III. Apply extraoral warm compress

121. Which of the following is correct to control mild symptoms that occur following sodium hypochlorite overflow into the periapical region during root canal irrigation?

A) I only

B) III only

C) I and II

D) II and III

E) I, II and III

122. In case of a file fracture in the canal during root canal treatment, which of the following pulpal or periapical pathologies has the least negative effect on the prognosis of the tooth?

A) Hyperplastic pulpitis

B) Necrotic pulp

C) Acute apical periodontitis

D) Chronic apical periodontitis

E) Chronic apical abscess

123. Which of the following has the most corrosive effect on stainless steel root canal instruments?

A) Sodium hypochlorite

B) Ethylenediamine tetraacetic acid

C) Chlorhexidine gluconate

D) Citric acid

E) Iodine potassium iodide

124. Which of the following chemicals is not preferred for softening gutta-percha?

A) Eucalyptol

B) Xylene

C) Orange oil

D) Halothane

E) Maleic acid

125. Which of the following is the most likely diagnosis for a tooth with pain during biting, tenderness during percussion, presence of a deep and narrow periodontal pocket during examination with a periodontal probe, and a fistula opening in the attached gingiva?

A) Vertical root fracture

B) Lateral luxation injury

C) Hyperplastic pulpitis

D) Chronic apical abscess

E) External cervical resorption

I. They are preferred because they have an elasticity value close to the elasticity modulus of dentin.

II. They can be easily detected on radiographs because they are radiopaque.

III. They are less likely to cause root fractures than screw-mounted metal posts.

126. Which of the above statements about carbon fiber posts are correct?

A) Only I

B) Only II

C) I and III

D) II and III

E) I, II and III

127. In which of the following should antibiotics be prescribed as supportive treatment after endodontic intervention?

A) Symptomatic irreversible pulpitis

B) Cellulitis

C) Chronic apical periodontitis

D) Necrotic pulp

E) Chronic apical abscess

I. Sodium hypochlorite is used because it is an organic tissue solvent and provides disinfection.

II. EDTA is used because it is a strong antibacterial agent.

III. 5.25% sodium hypochlorite irrigation releases growth factors from dentin.

IV. The 17% form of EDTA is preferred because it causes crown discoloration at high concentrations.

128. Which of the above statements are correct regarding disinfection procedures in regenerative endodontic treatments applied to young permanent teeth with necrotic pulp?

A) Only I

B) I and III

C) I and IV

D) II and IV

E) II and III

I. More than 50% of it dissociates into Ca and OH ions at body temperature.

II. It has high solubility in water.

III. Its pH is basic.

IV. In cases such as root resorption, an aqueous carrier is preferred.

V. Zinc oxide increases the durability of eugenol-based pastes.

129. Which is correct about Ca(OH)_2_?

A) I-III-IV

B) III-IV

C) III-IV-V

D) II-III-IV-V

E) I-II-III-IV-IV

I. Decrease in dentin permeability

II. Formation of repair dentin

III. Inflammation of the pulp connective tissue on the surface closest to the dentin

IV. Movement of fluid in the dentin tubules

130. Which of the above are the reactions of the pulp-dentin complex to protect the pulp against caries?

A) I

B) I – II

C) II – III

D) I – II – III

E) II- III – IV
